# Prognostic factors of successful tympanoplasty in pediatric patients: a cohort study

**DOI:** 10.1186/1471-2431-12-67

**Published:** 2012-06-12

**Authors:** Nuria Esperanza Boronat-Echeverría, Esmeralda Reyes-García, Yolanda Sevilla-Delgado, Héctor Aguirre-Mariscal, Juan Manuel Mejía-Aranguré

**Affiliations:** 1 Department of Pediatric Otorhinolaryngology, High Specialty Medical Care Unit of the Pediatric Hospital of the Centro Médico Nacional Siglo XXI, Instituto Mexicano del Seguro Social, Av. Cuauhtémoc 330, México City, 06720, Mexico; 2 Unit of Research in Clinical Epidemiology, High Specialty Medical Care Unit of the Pediatric Hospital of the Centro Médico Nacional Siglo XXI, Instituto Mexicano del Seguro Social, Av. Cuauhtémoc 330, México City, 06720, Mexico

**Keywords:** Tympanoplasty, Myringoplasty, Otologic surgical procedures, Otorhinolaryngologic surgical procedures, Epidemiologic methods

## Abstract

**Background:**

Tympanoplasty in children is a current and controversial theme. The success of tympanoplasty traditionally has been measured only by the post-operative integrity of the graft. Yet, there are other variables that may be used to determine success. The objectives of the present work were to analyze which factors are predictive of successful tympanoplasty in pediatric patients and to construct and validate a prognostic index that could be used as a tool to predict the success of tympanoplasty in children.

**Methods:**

**Setting.** Department of Pediatric Otorhinolaryngology, tertiary-care hospital, Mexico City. **Patients**. Forty-eight patients, who were older that five years of age, had persistent perforation of the tympanic membrane, and had undergone tympanoplasty (January 2005–June 2008), were followed for a year. **Main Outcome Measures.** The factors tested for their value as predictors were the following: age at time of surgery, state of contralateral ear, previous adenoidectomy, cause of perforation, size of perforation, infection at the time of surgery, state of mucosa, age at first occurrence of perforation, presence of craniofacial dysmorphia, and surgical technique. These factors were compared with the criterion, success, which was defined as attaining three positive outcomes: 1) integrity of the implant or membrane; 2) minimum of 10-dB gain in the auditory threshold or, in the case of normal hearing, conservation of same; and 3) air-filled space in the middle ear. The best model was obtained through logistic regression analysis; the model was validated.

**Results:**

The most balanced prediction model was that in which the three success criteria were included, with age, surgical technique, and infection at surgery being excluded as variables. The additional 12 pediatric cases used in the validation had a probability of success >0.425 (best cut-off level); two patients (17%) had poor evolution.

**Conclusions:**

This is the first study that validated a predictive index of the result of tympanoplasty in children. This index predicted 81% of the successful outcomes.

## Background

Tympanoplasty in children is a current and controversial theme [1,2]. Previously reported success rates for tympanoplasty in children have ranged between 56–94%, with this wide range being attributed to different selection criteria and definitions of success. The latter parameter traditionally has been measured only by the post-operative integrity of the graft [1-6]. Yet, there exist other valuable characteristics to consider, as it is known that children in general, and those who have undergone repair of the tympanic membrane, in particular, present a greater risk for retractions, serous otitis media, and re-perforation with episodes of acute otitis media [1]. In addition, with a pediatric patient, the surgery itself may be considered as being more difficult technically, due to the narrowness of the external ear canal and the generally smaller size of the ear, thus contributing to a poor result, but of a functional type. Therefore, over time, otorhinolaryngologists dedicated to pediatric pathology have considered it necessary to have a more complete definition of “success”—one that should include 1) integrity of the graft or membrane; 2) post-operative gain (minimum of 10 dB) in the auditory threshold, or conservation of hearing; and 3) complete healing, with the space of the aerated middle ear manifested by the graft located in the correct anatomical position, with neither atelectasis nor otitis media with effusion (OME) [7-10].

Arguments in favor of surgery at an earlier age (under 5 years) are the following: 1) reduction in the number of visits to the doctor, which are required for the follow-up of a minor with perforated eardrum; 2) hypoacusis and privation of aquatic activities with affect on the quality of life; 3) higher incidence of severe secondary complications due to chronic otitis media in younger children; 4) better cochlear reserve at younger ages, with greater potential to restore and preserve hearing; 5) limitation of the damage that chronic infection can cause to other structures in the middle ear; and 6) auditory loss that alters the development and the quality of academic activities [11-16]. Despite this arguments, the recommendation to delay tympanoplasty, generally until six years of age, is widely accepted. In support of said recommendation are the following aspects: alterations in the healing process or re-perforation of the graft by repetitive infections of the superior respiratory tracts; unpredictable function of the Eustachian tubes; immunological immaturity; possibility of spontaneous resolution; difficulty of post-operative care; lack of confidence of the part of the parents in the procedure; and equivalence of the perforation to the function of the ventilation tube [2,13,14]. There is evidence that tympanoplasty is more successful in children over six years of age [1,2,4,6,10,12-14], although other studies have not demonstrated significant differences in this respect [1,3,17].

In addition to age, other factors have been suggested as interfering with the success of the surgery: the size and site of the perforation; cause of the perforation; active infection at the time of surgery; the state of the auditory ossicles and of the mucosa of the middle ear; state of the contralateral ear (as a measure of the function of the Eustachian tube), and the surgical technique utilized [17]. The presence of adenoid tissue, whether it be hypertrophic or chronically infected, has been proposed as a prognostic factor; however, no statistically significant association has been demonstrated, above all in short-term results [1,2,4,18]. A factor that could have interfered in the result was the definition of success that was used. Children with craniofacial syndromes, with or without cleft palate, present a different evolution; for these children, the results reported in the literature are controversial [19,20].

The objectives of the present work were to analyze which factors are predictive of success of tympanoplasty in pediatric patients and to construct and validate a prognostic index that could be used as a tool to predict the success of tympanoplasty in children.

## Methods

### Study design

Prospective cohort. All patients were treated in the Department of Pediatric Otorhinolaryngology, High Specialty Medical Care Unit of the Pediatric Hospital of the Centro Médico Nacional Siglo XXI, one of the two tertiary-care hospitals of the Instituto Mexicano del Seguro Social (IMSS) in Mexico City. IMSS is one of the largest institutions of social security and health in the world, the largest in Latin America, and the most important in Mexico: 35 million people have the right to receive attention from IMSS [21]. This study was approved by the Ethics Board of the Pediatric Hospital of the Centro Médico Nacional Siglo XXI (R-2009-3603-12). Written informed consent for participation in the study was obtained from the participants’ parents.

### Patient population

For inclusion in this study, the pediatric patients had 1) to be older than five years of age; 2) to have had persistent uni- or bilateral perforation of the tympanic membrane, defined as perforation of the eardrum, lasting more than three months, which was caused by trauma, infection, or placement of ventilation tubes; and 3) to have undergone tympanoplasty (onlay or underlay) in the Department of Pediatric Otorhinolaryngology of the Pediatric Hospital, by whatever technique, during the period January 2005 and June 2008, with a minimum follow-up of one year. Excluded from this cohort were patients with previous failed tympanoplasties and/or with associated chronic rhinosinusitis, uncontrolled respiratory allergy, obstructive septal deviation, congenital or acquired immunodeficiency, or other factors that alter immunity. The size of the cohort was 59 patients, of which 11 were eliminated (eight were excluded because ossicular chain was affected, according to preoperative audiometry; three were excluded due to files being lost during follow-up). Therefore, the results of 48 patients (29 males; 19 females) were analyzed. The variables analyzed for their predictive value were the following: age at surgery (>6 and ≤6 years); state of contralateral ear (without otitis media vs. with otitis media); previous adenoidectomy (no vs. yes); cause of the perforation (infection vs. post implantation of the ventilation tube); size of the perforation (<50% vs. >50%); active ear infection at the time of surgery manifested by otorrhea (present vs. absent); state of the mucosa (normal vs. edema and/or polypoid mucosa); age at onset of chronic otitis media; presence of craniofacial dysmorphias (present or absent); and surgical technique used (onlay vs. underlay) (Table 1). These variables were compared to the criteria of success outcomes: 1) closure of the perforation (yes vs. no); 2) gain of at least 10 dB in the threshold of hearing [22] with respect to the pre-operative value, or conservation of hearing level in the case of normal pre-operative hearing; and 3) complete healing with the air-filled, middle-ear space expressed by an implant in anatomical position, without tympanic membrane atelectasis or OME (Table 2).

**Table 1:** Distribution frequency of non-continuous predictive variables in relation to the outcome of tympanopalsty before regression analysis

**Variable**	**Outcome**				
	**Success**		**Failure**		***P***
	**n=27**	**%**	**n=21**	**%**	
**Contralateral ear**					
Normal	18	66.7	16	76.2	0.47
Tympanic perforation/OME^a^/atelectasis	9	33.3	5	23.8	
**Previous adenoidectomy**					
No	15	55.6	12	57.1	0.91
**Cause of perforation**					
Infection	23	85.2	18	85.7	0.95
**Size of perforation**					
<50%	15	55.6	14	66.7	0.43
**State of mucosa**					
Normal	23	85.2	17	81.0	0.70
**Surgical technique**					
onlay	23	85.2	18	85.7	0.96
**Craniofacial dysmorphia**					
No	22	81.5	21	100.0	0.04
**Otorrhea at time of surgery**					
Absent	26	96.3	19	90.5	0.40

^a^OME: otitis media with effusion. For simplicity the positive results are showed. Negative variables are the complement of the showed number.

**Table 2:** Distribution of outcome variables for tympanoplasty

**Variable**	**Patients**	
	**n**	**(%)**
**Closure of perforation**		
Yes	45	93.8
No	3	6.3
**Hearing gain**		
Conserved or gain (10–20 dB)	32	66.7
No gain	16	33.3
**Air-filled space**^**a**^		
Adequate	39	81.2
Otitis media with effusion	4	8.3
Partial atelectasis	2	4.2
**Success (all three variables)**	**27**	**56.3**

^**a**^In three cases, re-perforation occurred and, therefore, these cases are not included.

### Analysis

A logistic regression model was constructed in which all the predictive variables were included. With the modelling, the βs were utilized to develop a predictive model of the evolution for the patients. In this type of analysis, the clinical relevance is more important than are the P values; therefore, the predictive models must be validated [23]. Once the model had been developed, we proceeded to validate it with the inclusion of 12 new patients, for each of whom the variables of the partial model and the probability obtained were compared with the result of the evolution of the patient during a one-year follow-up. To identify the best value of prediction, a receiver operating characteristic curve was constructed (data not shown). It was thereby determined that the probability of 0.425 had the best sensitivity and the best specificity (81.5% and 66.7%, respectively). All analyses and formulas were conducted by using the SPSS v.15 program (SPSS, Inc., Chicago, IL, USA).

## Results

The distribution of the outcome variables is shown in Table 1. When only the closure of the perforation was considered, the surgery could be considered successful in >90% of the cases; however, when the definition of success incorporated all three outcome variables (closure, hearing, and air-filled space), the success rate fell to 56.3%. The contralateral ear was normal in 18 of 27 patients with successful outcome and in 16 of 21 patients with failed outcome (P = 0.47). Tympanic perforation/OME/atelectasis was present in nine patients with successful outcome and in five with failed outcome (P = 0.91). Children without craniofacial dysmorphia were among the patients with either successful or failed results, 22 and 21 respectively; this difference was statistically significant (P = 0.04). These results showed the difficulty in using only one factor to predict a successful outcome after tympanoplasty (Table 2).

Only five patients (10.4%) presented craniofacial dysmorphias; three cases (6.2%) corresponded to sequelae of uni- or bilateral cleft lip or cleft palate, and two (4.1%) to submucosal cleft palate (data not shown). The median age of the group with successful outcome was 11 years of age (range: 5–16 years), whereas that of the group with a failed outcome was nine years of age (range: 6–15 years). The median age of onset of symptoms was five years of age for both the successful- and failed- outcome groups (range: 1–15 years and 1–7 years, respectively).

The values of the β coefficients are shown in Table 3. Because they did not represent differences, the following three variables were excluded from the partial model: age at time of surgery, surgical technique (onlay vs. underlay), and the presence of otorrhea at the time of surgery

**Table 3:** Logistic regression to identity the variables that predict the success of tympanoplasty

**Predictive variable**		**Regression parameter**^**a**^
Code	Description	β
1	Absence of craniofacial dysmorphias	-0.812
2	State of contralateral ear	0.777
3	Previous adenoidectomy	-0.123
4	Cause of perforation	-0.319
5	Size of perforation	0.659
6	State of mucosa	-0.689
7	Age at onset of symptoms	-1.330

^a^Constant: 0.543; the P values are not important in predictive models.

Although all 12 patients included in the validation had a probability of success >0.425 (the value selected as being best for the cut-off level), two patients (16.7%) had a poor evolution (Table 4). The values of the validation were obtained in the following manner (data from the first patient (Table 4) is used in the example): the onset of symptoms was after six years of age, corresponding to a value of 1 for the variable x_1_ (Table 4); this value was multiplied by the value of the corresponding coefficient β for that variable (onset of symptoms) in Table 3, i.e., β = −1.330. For the variable, contralateral ear, the patient’s ear was normal, giving a value of 1, which was then multiplied by the value of β for contralateral ear (0.777). For the variable, adenoidectomy, the multiplication product, 1 × -0.123, corresponded to the fact that the patient had undergone adenoidectomy prior to the tympanoplasty, with −0.123 being the coefficient for adenoidectomy in the model. Substituting the products into equation of probability in logistic regression, the resulting value was 0.78.

**Table 4:** Results of validation study performed after construction of prognostic index from logistic regression model

**Predictive variable**		**Patient number**											
Code	Description	**1**	**2**	**3**	**4**	**5**	**6**	**7**	**8**	**9**	**10**	**11**	**12**
1	Absence of craniofacial dysmorphias	1	0	1	1	1	1	1	1	1	1	1	1
2	State of contralateral ear	1	0	1	0	1	0	1	1	1	1	1	1
3	Previous adenoidectomy	1	0	1	0	0	0	0	0	0	0	0	0
4	Cause of perforation	1	1	0	0	1	0	1	0	0	0	1	0
5	Size of perforation	1	0	0	1	1	0	0	1	1	1	0	1
6	State of mucosa	1	1	1	1	1	1	1	1	1	1	1	1
7	Age at onset of symptoms	1	1	0	0	1	0	0	1	1	1	1	1
Probability of success		0.78	0.85	0.57	0.57	0.76	0.72	0.62	0.70	0.70	0.70	0.86	0.70
Outcome		**S**^a^	**S**	**S**	**S**	**S**	**S**	**F**^b^	**F**	**S**	**S**	**S**	**S**

^a^S: successful outcome.

^b^F: failed outcome.

## Discussion

The principal results of the validation study showed that the variables that best predicted the success of tympanoplasty were the following seven: the age at onset of symptoms (>6 years); the state of the contralateral ear (normal); prior adenoidectomy; the cause of perforation (trauma/implantation of ventilation tube); size of the perforation (<50%); state of the mucosa (normal); and the absence of craniofacial dysmorphias. By incorporating a more functional definition of success (i.e., measured by means of three criteria: integrity of graft, hearing, and adequate air-filled space in the middle ear), the rate of success fell to 56.3%, compared to that (93.8%) found when only the integrity of the implant or closure of perforation was considered (Table 2). This is not an isolated finding for pediatric patients. Bluestone et al. [24] published a success rate of 35%; Manning et al. [16] reported 78% success for integration of the graft, but only 52% showed adequate function of the Eustachian tube. In comparison, data for adults show that closure of the perforation is achieved in approximately 80% to 90% of cases; however, only 70% have an intact tympanic membrane, without imperfections such as pockets of retraction or lateralization of the graft, so that different degrees of improvement in the auditory threshold are found in 67.2%, with improvement of >20 dB (16.4%) in three-year follow-ups [10]. On this point, it is worth mentioning that the most acceptable definition of success in auditory gain is the closure of the air-bone gap, resulting in a gain of ≥20 dB [22]. Therefore, in this study, the auditory component of success was defined as a gain in auditory threshold of 10–20 dB, or more, and not as closure of the air-bone gap, the reason being that the latter is measured only by tonal audiometry, which was not possible to do with all the children in this study; we had technical problems with the children. For those cases in which tonal audiometry could not be done, those patients were assessed by using brainstem auditory evoked potentials.

To our knowledge, this is the first study to validate an index that predicts the result of tympanoplasty in children. In general terms, the characteristics of the patients were similar to those treated in other hospitals. For the population of the United States, the most frequent causes of perforation have been, first, a result of the placement of ventilation tubes and, second, chronic perforation due to infection [4]. However, other reports have mentioned a high frequency of perforations with infectious etiology [16], the frequency being equal to that reported for patients in this series.

It is important to point out that, in the present work, age at the time of surgery was excluded because this variable did not show sufficient weight in the model to predict the success of surgery. A limitation of this study would be the considering that the youngest patient included was five years of age. Using logistical regression analysis, Sckolnick et al. [3] postulated that, as age increased, the odds ratio of success diminished, with the cut-off point placed at nine years of age; that is, the best prognosis was for 1–5 year-old patients, declining 9% for each year of age thereafter, until nine years of age when the success rate increased to a value similar to that found for those older than 16 years of age. For this reason, it is possible to identify a group of patients, 7–12 years of age, with a lower rate of success; the majority of the population studied was, on average, 7.1 years old [3]. The present study is one of the few that evaluate the results of tympanoplasty in very young children. A study by Black et al. in 1994 included patients as young as two years of age; no statistically significant differences were encountered in the results of the surgery for these children [11]. In the only meta-analysis that individually evaluated articles that were cited in the current study, the authors showed that, in the majority (25 of 30) of these reports, no relation was found between age and the success of surgery. This contrasts with the results of the same meta-analysis, in which a statistically significant relation was found between this factor and a successful result. The authors of this meta-analysis attributed these discrepancies to the following: the majority of the individual studies analyzed either did not include patients younger than seven years of age, used a different definition of success, or had methodological errors, such as the size of the sample or the type of study (retrospective) [1]. In a study published in 2010, which reported (retrospective) the results of 132 tympanoplasties performed on children, ranging from 6–15 years and divided into two groups (<8 and >8 years), once again no statistically significant differences between age and successful result were found [18]. It is of note that, in that study, the definition of success was the same as the one used in the present study; one limitation of this definition is that the frequency of success is lower than when just one factor is considered.

Another important point to consider is the age of onset of chronic otitis media. In the literature, this is used in the age of the perforation. For example, patients with retained ventilation tube, who underwent tympanoplasty during the removal of the tube and who did not suffer changes due to chronic inflammation are said to be at age “zero” of the perforation. This population differs from those whose perforation occurred either after a process of otitis, or secondary to the extrusion of a tube, but for whom a determined period of time passed before the tympanoplasty was performed [4]. In that same study, the authors found a statistically significant relation between the age at which the ear was perforated and the result of the surgery; nevertheless, upon excluding the patients at age zero of the perforation, this relation loses significance. In other studies, no relation was found between these factors [1,4,9]. The sample size of our study was small; however, the age of onset of chronic otitis media was associated with the success of tympanoplasty.

For patients with craniofacial dysmorphias (basically, the sequelae of cleft lip or cleft palate), the results of tympanoplasty are controversial. On one hand, Dornhoffer et al., in a series of 20 patients, with a total of 26 otological surgeries, concluded that tympanoplasty is a reasonable treatment for patients with sequelae of cleft lip or cleft palate. In that work and in the current study, the same three criteria of success were used and similar results were obtained [25]. In a retrospective study of 26 patients who had sequelae to cleft lip or cleft palate and who underwent tympanoplasty, no statistically significant difference was found in the results for integration of the graft, hearing, and the necessary equalizing of pressure in the Eustachian tube, as compared with the results for patients of the same age without cleft palate [21]. Despite the small sample size in our study, this variable was related with the prognoses of the patients, one reason in this study would be that the increased technical difficulty encountered in performing tympanoplasty on patients with craniofacial dysmorphia tends to negatively affect the outcome of the surgery.

One limitation of this study was that, with the seven variables included in the predictive model, it was possible to predict only 81% of the successful outcomes. In the validation study, all patients with a successful evolution could be predicted. Nevertheless, the specificity was only 67%; therefore, it was expected that this model could not predict 33% of the failures. In the present study, 17% of the patients who had been predicted to have successful outcomes had, in fact, unfavorable post-surgical evolutions. However, the predictive model produced better results than did analyzing each variable separately. The model that included the three outcome criteria was the most equilibrated of these models was the one that incorporated all three parameters of success as the outcome criterion, because it predicted a greatest number of failures without affecting the percentage of predicted successes.

As mentioned above, the sample size of this study is its main limitation; thus, it will be necessary to include a greater range of patients in order to evaluate the benefit of the prognostic index presented here. As indicated by Sackett et al. [23], it is preferable to quantify the probability of success of a therapeutic intervention when explaining the intervention to a patient or to the patient’s family members, because a calculated value provides them with a more objective tool with which to make a decision as to whether an intervention is appropriate or not.

## Conclusions

The proposed model provides the pediatrician and the otolaryngologist a tool with which to quantify the probability of success that a patient, who presents with chronic perforation of the tympanic membrane and requires surgical intervention, will have.

## Competing interests

The authors declare that they have no competing interests.

## Authors’ contributions

All five authors conceived the study, participated in the design and coordination of the study, and helped to draft the manuscript. NEBE and JMMA performed the statistical analysis. All authors read and approved the final manuscript.

## Pre-publication history

The pre-publication history for this paper can be accessed here:

http://www.biomedcentral.com/1471-2431/12/67/prepub
